# Editorial: Integrating transcriptional modulation in systemic tumor therapy

**DOI:** 10.3389/fonc.2024.1385766

**Published:** 2024-02-28

**Authors:** Daniel Heudobler, Florian Lüke, Lina Ghibelli, Albrecht Reichle

**Affiliations:** ^1^ Department of Internal Medicine III, Hematology and Oncology, University Hospital Regensburg, Regensburg, Germany; ^2^ Bavarian Cancer Research Center (BZKF), University Hospital Regensburg, Regensburg, Germany; ^3^ Department of Biology, University of Rome Tor Vergata, Rome, Italy

**Keywords:** pioglitazone, interferon-α, dexamethasone, all-trans retinoic acid, tumor tissue editing, phenotypic plasticity, anakoinosis, low dose metronomic chemotherapy

Nuclear receptor antagonists for transcriptional reprogramming of tumor tissues are a success story in prostate and breast cancer (1). Nuclear receptor agonists, such as glucocorticoids, play a key role in therapies for hematologic malignancies.

Nuclear receptor agonists’ context-dependent activity profiles, theme-dependent interactions with one another, and often poor monoactivity are reasons for their hesitant combinatorial application in oncology (2, 3). Harrer et al. present novel therapy approaches unlocking the phenotypic plasticity of tumors as the basis for the comprehensive implementation of nuclear receptor agonists in tumor therapy (4, 5).

Nuclear receptors ubiquitously balance normal and malignant tissue homeostasis and show differential cell type- and tumor stage-dependent expression patterns, varying upon microenvironmental triggers (6). At first sight, less fixable functional receptor constellations do not invite combinatorial use in relapsed/refractory (r/r) neoplasias (4).

Therapeutic targeting of the tumors’ phenotypic plasticity necessitates the reprogramming of cancer hallmarks into biologic hallmarks attenuating tumor growth. The intention to phenotypically edit tumor tissues, in analogy to genetic tumor editing, enabled the repositioning of nuclear receptor agonists in dual or triple combinations to unlock the phenotypic plasticity of neoplasias, resulting in long-term control of malignancies in the r/r stage (4).

For differentially unlocking tumor phenotypes with transcriptional modulators, low dose metronomic chemotherapy emerged as a prerequisite in non-oncogene addicted tumors (7). Metronomic chemotherapy tightens phenotypic tumor heterogeneities by altering tumor stress response. Tightened phenotypic tumor heterogeneity facilitates clinically relevant combined transcriptional modulation with pioglitazone, a peroxisome-proliferator activating receptor (PPAR)α/γ agonist, and histology-dependent, all-trans retinoic acid (ATRA), dexamethasone, or cytokines (interferon-α) for establishing novel homeostatic balances that overcome post-therapy genetic tumor heterogeneity, metastases, resistance, and tumor cell repopulation (M-CRAC) in r/r neoplasias and at best induce CR or cCR in hematologic neoplasias, cancers and sarcomas (4).

Thus, the M-CRAC obstacle, a frequently occurring phenomenon following any pulsed systemic therapy using maximum tolerable doses, approaches a therapy-technical solution with tumor tissue editing techniques. A cure in the r/r stage is within reach, as shown (4).

**Figure 1** indicates how novelly implemented biomodulatory treatment elements may on-topic edit tumor tissues for M-CRAC control, CR, and cCR induction, and how the drugs discussed in this Research Topic might contribute to tumor tissue editing.

**Figure 1:**
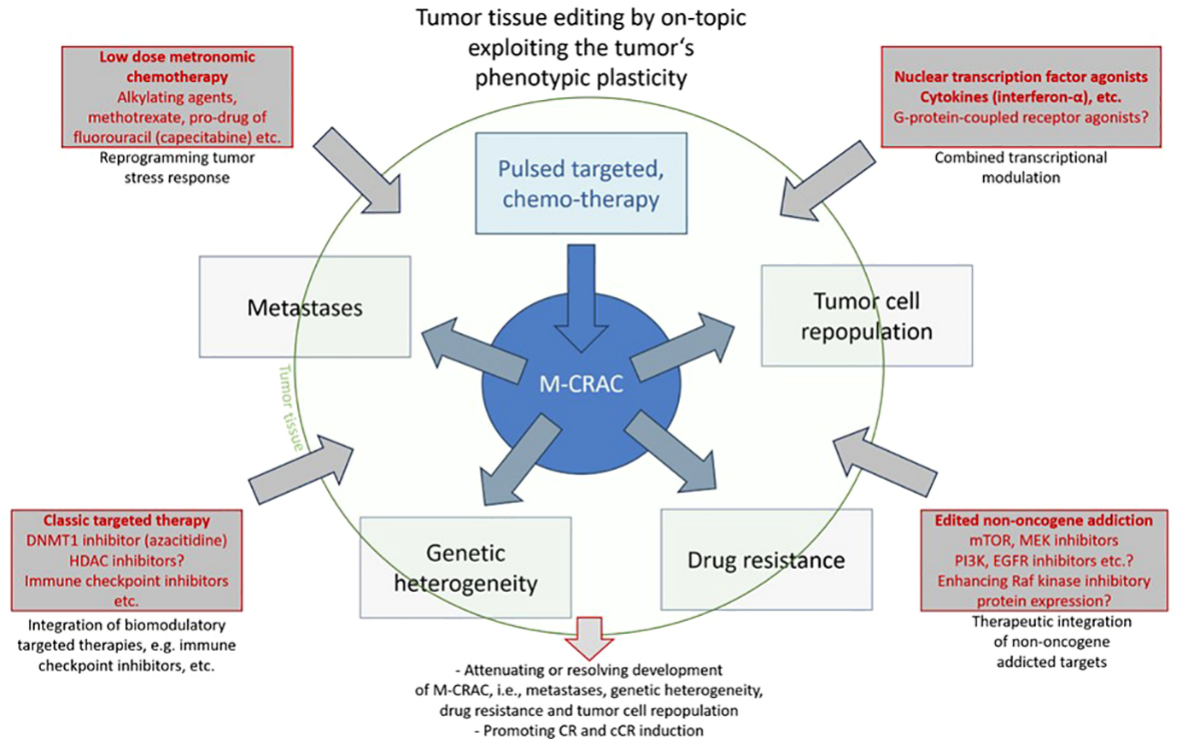
Tumor tissue editing by on-topic exploiting the tumor’s phenotypic plasticity.

The case report by Reuthner et al. on a heavily pre-treated patient with r/r Hodgkin’s lymphoma (HL) is a further cornerstone illustrating the qualities of tissue editing techniques in r/rHL. Firstly, irrespective of the kind of pre-treatment, chemotherapy, high-dose chemotherapy with autologous or allogeneic peripheral blood stem cell transplantation, or approved immunotherapies, such as immune checkpoint inhibitors, tissue editing may induce cCR in r/rHL (8). Secondly, a crucial additional element of the editing therapy, here metronomic chemotherapy, pioglitazone, dexamethasone, and etoricoxib, is everolimus. The editing technique in r/rHL facilitates a strong non-oncogene addiction to the mammalian target of rapamycin (mTOR). mTOR inhibitors have some monoactivity in r/rHL in combination with pulsed chemotherapy even with no beneficial impact (9, 10).

The paper by Mock et al. gives hints that inhibition of the upstream part of the mTOR pathway, the phosphoinositide 3-kinase (PI3K), in addition to epidermal growth factor (EGFR) inhibition could be a successful way to control respective activating mutations or tumor-associated up-regulation in human papillomavirus negative squamous cell head and neck cancers (HNSCC). A recent randomized trial for r/r HNSCC showed that tissue editing with low dose nivolumab, an immune checkpoint inhibitor, plus low-dose metronomic chemotherapy, cyclooxygenase-2, and EGFR inhibitor significantly improves survival compared to the same schedule without nivolumab (11). Again, on-topic reprogramming cancer hallmarks, here combined reconstitution of immunosurveillance, is highly efficacious in r/r disease.

Histone deacetylase inhibitors (HDACi) could play an important role in the therapy of acute lymphocytic leukemia (ALL), as reviewed by Zhang et al. HDACi and DNA (cytosine-5)-methyltransferase 1 inhibitors, such as azacitidine, alter clinically relevant epigenetic scenery, thereby providing access to the successful use of nuclear receptor agonists. Low-dose azacitidine plus triple transcriptional modulation with pioglitazone and ATRA facilitates functionally relevant differentiation of acute myelocytic leukemia (AML) blasts in r/r non-promyelocytic leukemia (non-PML) AML (4, 12).

As shown by Tan et al., a prognostically relevant expression pattern of G-protein-coupled receptors in osteosarcoma could provide novel targets for tissue editing with approved but repurposed drugs. Inhibition of G-protein-coupled receptor kinase 2 attenuates malignant cell growth by downregulation of the IGF1 receptor (13).

Lai et al. address the pathophysiology of M-CRAC development. Aggressive breast cancer down-regulates metastasis suppressing Raf kinase inhibitory protein (RKIP) and may promote PPARγ activation (14). RKIP inhibits many kinases, Raf/Mek/Erk, and NF-kB-dependent pathways and regulates epithelial mesenchymal transition (15). MEK inhibition plus pioglitazone engages tumor plasticity and differentiates cancer cells to adipocytes in animal models (16). Pioglitazone-related inflammation control is an important partial aspect of the presented editing therapies for r/r neoplasias (4, 16).

Dependent on the transcriptionally active combination partners, dual or triple transcriptional modulation facilitates on-topic reprogramming of tumor-associated cancer hallmarks into biologic hallmarks controlling tumor growth, such as differentiation (pioglitazone, ATRA), inflammation control (pioglitazone, interferon-α, dexamethasone), enhanced immunosurveillance (pioglitazone, metronomic chemotherapy), reprogrammed metabolism (pioglitazone), and alternative tumor cell death pathways (4). Histologically different neoplasias share access to clinically important targets suitable for reprogramming hallmarks of cancer with nuclear receptor agonists/cytokines (4). As a novel anti-cancer drug, pioglitazone provides tumor-type agnostic efficacy across thirteen histologically different neoplasias and may resolve cachexia (5).

Tightening phenotypic tumor heterogeneity, here by including low-dose metronomic chemotherapy or epigenetic modulators, enhances the clinical activity of a huge repertoire of drugs concertedly targeting tumor and stroma cells, as exemplarily shown for transcriptional modulators, and provides structured access for rescuing r/r neoplasias (**Figure 1**). Poor monoactivity of the applied drugs, even in the case of metronomic chemotherapy, due to scheduled dose reductions, underlines their reprogramming activity profile, termed anakoinosis. Correspondingly, toxicity profiles are tolerable (4).

Successful tumor tissue editing, including agonists of nuclear receptors/cytokines, fixes M-CRAC as a general therapeutic problem arising following pulsed systemic tumor therapy by providing multifold on-topic solutions to resolve M-CRAC. Tumor tissue editing demonstrates unique accessibility to M-CRAC, just in the most frequently occurring tumors with missing driver mutations or in case of complex chromosomal aberrations, e.g., in r/r non-promyelocytic AML (4, 12).

## Author contributions

DH: Writing – original draft, Writing – review & editing. FL: Writing – original draft, Writing – review & editing. LG: Writing – original draft, Writing – review & editing. AR: Writing – original draft, Writing – review & editing.
